# Concomitant granule cell neuronopathy in patients with natalizumab-associated PML

**DOI:** 10.1007/s00415-015-8001-3

**Published:** 2016-01-25

**Authors:** Martijn T. Wijburg, Dorine Siepman, Jeroen J. J. van Eijk, Joep Killestein, Mike P. Wattjes

**Affiliations:** Department of Neurology, MS Center Amsterdam, VU University Medical Center, De Boelelaan 1117, 1081 HV Amsterdam, The Netherlands; Department of Radiology and Nuclear Medicine, MS Center Amsterdam, VU University Medical Center, Amsterdam, The Netherlands; Department of Neurology, Erasmus MC, University Medical Center Rotterdam, MS Center, Rotterdam, The Netherlands; Department of Neurology, Jeroen Bosch Hospital, ‘s-Hertogenbosch, The Netherlands

**Keywords:** Multiple sclerosis, Granule cell neuronopathy, PML, Cerebellar atrophy, MRI

## Abstract

Granule cell neuronopathy (GCN) is a rare JC virus infection of the cerebellar granule cell neurons in immunocompromised patients. On brain imaging, GCN is characterized by cerebellar atrophy which can be accompanied by infratentorial white matter lesions. The objective of this study is to investigate the prevalence of MRI findings suggestive of GCN in a large natalizumab-associated progressive multifocal leukoencephalopathy (PML) cohort. MRI scans from before, at the time of, and during follow-up after diagnosis of PML in 44 natalizumab-treated MS patients, and a control group of 25 natalizumab-treated non-PML MS patients were retrospectively reviewed for imaging findings suggestive of GCN. To assess and quantify the degree of cerebellar atrophy, we used a 4 grade rating scale. Three patients in the PML group showed imaging findings suggestive of GCN and none in the control group. In two of these PML patients, cerebellar atrophy progressed from grade 0 at the time of diagnosis of isolated supratentorial PML to grade 1 and 2 after 2.5 and 3 months, respectively, in the absence of infratentorial white mater lesions. The third patient had grade 1 cerebellar atrophy before diagnosis of infra- and supratentorial PML, and showed progression of cerebellar atrophy to grade 2 in the 3 months following PML diagnosis. None of the other eight patients with infratentorial PML lesions developed cerebellar atrophy suggestive of GCN. Three cases with imaging findings suggestive of GCN were detected among 44 natalizumab-associated PML patients. GCN may, therefore, be more common than previously considered in natalizumab-associated PML patients.

## Introduction

Progressive multifocal leukoencephalopathy (PML) is a lytic infection of astrocytes, oligodendrocytes and neurons caused by the JC virus (JCV), leading to irreversible demyelination and neuronal damage [1]. PML occurs almost exclusively in immunosuppressed patients, and traditionally PML was mainly observed in human immunodeficiency virus (HIV)-infected patients and patients with hematologic malignancies. However, in recent years PML has emerged as a serious complication of immunosuppressive treatment. In particular, PML associated with monoclonal antibody treatment of autoimmune diseases has attracted major attention. PML has been reported especially in association with the use of natalizumab, a monoclonal antibody against the α4-integrin adhesion molecule approved for the treatment of active relapsing–remitting multiple sclerosis (MS) [2, 3]. As of June 3, 2015, 566 PML cases have been reported in more than 138,800 natalizumab-treated MS patients [4].

In addition to PML affecting white matter and gray matter cells leading to heterogeneous imaging findings [5], JCV-associated diseases sparing white matter cells and almost exclusively affecting gray matter cells have been described, such as JCV encephalopathy involving the cortical gray matter and granule cell neuronopathy (GCN) [6]. GCN is characterized by a JCV infection of the cerebellar granule cell neurons causing neuronal loss and gliosis, ultimately leading to cerebellar atrophy and cerebellar neurological symptoms [7, 8]. Even though cerebellar atrophy is the leading imaging sign in the vast majority of GCN patients, the imaging features may be heterogeneous and subtle. The progression of cerebellar atrophy can be accompanied by white matter changes of the cerebellum and sometimes in the brainstem [9]. Involvement of the brainstem in GCN patients can result in linear white matter hyperintensities in the pons similar to those seen in multiple system atrophy (MSA) and in some PML patients, which coined the term “hot cross bun sign” [9–11]. GCN has mostly been reported in immunocompromised HIV-infected patients. Recently, GCN has been described in two natalizumab-treated MS patients [12, 13]. GCN can develop either as isolated JCV-associated disease or concomitant to PML [8, 14].

Histopathological data have demonstrated that granule cell neurons are frequently infected in patients classified as PML, even in the absence of lesions suggestive of PML in the neighboring cerebellar white matter [15]. However, to date it is unknown to what extent infection of granule cell neurons leads to imaging signs of GCN in PML patients. In contrast to PML which can be detected in asymptomatic stages [16], GCN may escape early detection, especially in MS patients as they may have different stages of cerebral atrophy, both supra- and infratentorial, resulting from neurodegeneration in MS [17]. Underdiagnosis may thus easily occur, even in closely monitored patients like natalizumab-associated PML patients according to recent expert guidelines [18].

The aim of our study was to investigate the prevalence of imaging findings suggestive of GCN in a large cohort of natalizumab-associated PML patients. The second aim was to investigate a possible link between PML manifestations in the posterior fossa and the development of imaging findings suggestive of GCN.

## Patients and methods

### Patient selection, and patient consent

This was a retrospective study, reviewing brain MRI scans of natalizumab-associated PML patients for imaging findings suggestive of GCN. A total of 61 patients were included from two previously described datasets (17 patients from the Dutch–Belgian natalizumab-associated PML cohort and 44 patients who were referred by other institutions to our center for second opinion and research purposes) [19, 20]. For this study, the following inclusion criteria have been applied: (1) a diagnosis of natalizumab-associated PML according to the American Academy of Neurology (AAN) PML diagnostic criteria as definite, probable or possible PML [21]. (2) Availability of a brain MRI prior to, at the time of, and following PML diagnosis. (3) Availability of multiple brain MRI sequences, including at least T2 and/or fluid attenuated inversion recovery (FLAIR) weighted and T1-weighted sequences. (4) Imaging materials available in the Digital Imaging and Communication in Medicine (DICOM 3) file format. Exclusion criteria were: (1) insufficient follow-up MRIs (defined as no follow-up MRI available more than 3 months after diagnosis), to confidently determine the presence or absence of imaging findings suggestive of GCN. (2) Poor image quality impairing the assessment of cerebellar atrophy (i.e., movement artefacts, bad repositioning, etc.).

We randomly selected a control group of 25 patients from our local cohort of natalizumab-treated MS patients. Inclusion criteria for the control group were: availability of at least three serial brain MRIs during a follow-up period of approximately 1 year, treatment with natalizumab during the follow-up period without interruptions, at least 1 year of natalizumab treatment at the start of the follow-up period, and the absence of PML lesion development.

Written informed consent for the use of clinical and imaging data for research and educational purposes was obtained from all participants and the study was conducted with adherence to the ethical standards laid down in the 1964 Declaration of Helsinki and its later amendments.

### MRI protocols

The image acquisition parameters including pulse sequences, head coils and magnetic field strengths (1.5 and 3 T) and parameters related to spatial resolution were heterogeneous and based on local MRI protocols as the imaging data were collected from different centers. All patients had T2/PD-weighted images and/or FLAIR images available, and a T1-weighted sequence available.

### Image analysis and interpretation

Two raters with expertise in diagnosis and treatment of GCN and PML (MPW, MTW) analyzed all brain MR images in consensus on a digital workstation. As cerebellar atrophy is the main imaging finding in GCN, the reference MRI scan prior to PML diagnosis (defined as baseline scan), the MRI scan at diagnosis and the MRI scans during follow-up after PML diagnosis were analyzed with respect to the degree of cerebellar atrophy [8, 9]. We assessed and semi-quantified the extent of cerebellar atrophy based on a four-grade visual rating scale: grade 0: no atrophy; grade 1: dilated sulci; grade 2: loss of volume; grade 3: end-stage atrophy. This rating scale was derived in a modified form from visual rating scales for cortical atrophy assessment [22, 23]. Only those patients in whom cerebellar atrophy increased at least one grade at the time of, or after the onset of PML, were considered suggestive of GCN because there were not sufficient imaging studies available to assess the possible development of GCN before the baseline scan. Furthermore, we scored and classified the presence of infratentorial and supratentorial PML lesions at the time of PML diagnosis and during follow-up.

In the control group the same assessment and semi-quantification of cerebellar atrophy was performed for all MRI scans, at least three per patient, during the approximate follow-up period of 1 year (median follow-up: 13 months, range 11–15 months).

## Results

### Patients

Of the 61 collected natalizumab-associated PML patients, 44 met the inclusion criteria and were, therefore, included in this study (all 17 patients from the Dutch–Belgian natalizumab-associated PML cohort, and 27 patients who were referred by other institutions to our center for second opinion and research purposes). The demographic and clinical information of these patients and of the control group is presented in Table 1.

**Table 1:** Demographic and clinical information of the study participants

	PML group, *n* = 43 Median (range)	Control group, *n* = 25 Median (range)
Gender (female/male), *n* (%)	30 (68.2)/14 (31.8)	18 (72)/7 (28)
Age (years)	43.5 (32–71)	44.6 (21–52)
Natalizumab treatment duration (doses)	35.0 (13–86)	35.9 (12.1–88.4)
Asymptomatic at diagnosis, *n* (%)	8 (18.2)	n/a
Symptomatic at diagnosis, *n* (%)	36 (81.8)	n/a
Solely supratentorial PML lesions	35 (79.5)	n/a
Both supra- and infratentorial PML lesions	8 (18.2)	n/a
Solely infratentorial PML lesions	1 (2.3)	n/a

*PML* progressive multifocal leukoencephalopathy, *n/a* not applicable

### Baseline cerebellar atrophy

In 30 of the 44 patients in the PML group there was already a certain degree of cerebellar atrophy present at baseline (grade 0: 14 patients, grade 1: 24 patients, grade 2: 6 patients, grade 3: 0 patients). Also in the control group the majority of patients had pre-existing cerebellar atrophy (grade 0: 9 patients, grade 1: 13 patients, grade 2: 3 patients, grade 3: 0 patients). The degree of cerebellar atrophy of all study participants at baseline is shown in Table 2.

**Table 2:** Degree of cerebellar atrophy on brain MRI at baseline

	PML group, *n* = 44 (%)	Control group, *n* = 25 (%)
Cerebellar atrophy grade 0	14 (31.8)	9 (36)
Cerebellar atrophy grade 1	24 (54.5)	13 (52)
Cerebellar atrophy grade 2	6 (13.6)	3 (12)
Cerebellar atrophy grade 3	0 (0)	0 (0)

Four-grade cerebellar atrophy rating scale: grade 0: no atrophy; grade 1: dilated sulci; grade 2: loss of volume; grade 3: end-stage atrophy

### Imaging findings suggestive of GCN and clinical symptoms of cerebellar pathology

**Fig. 1:**
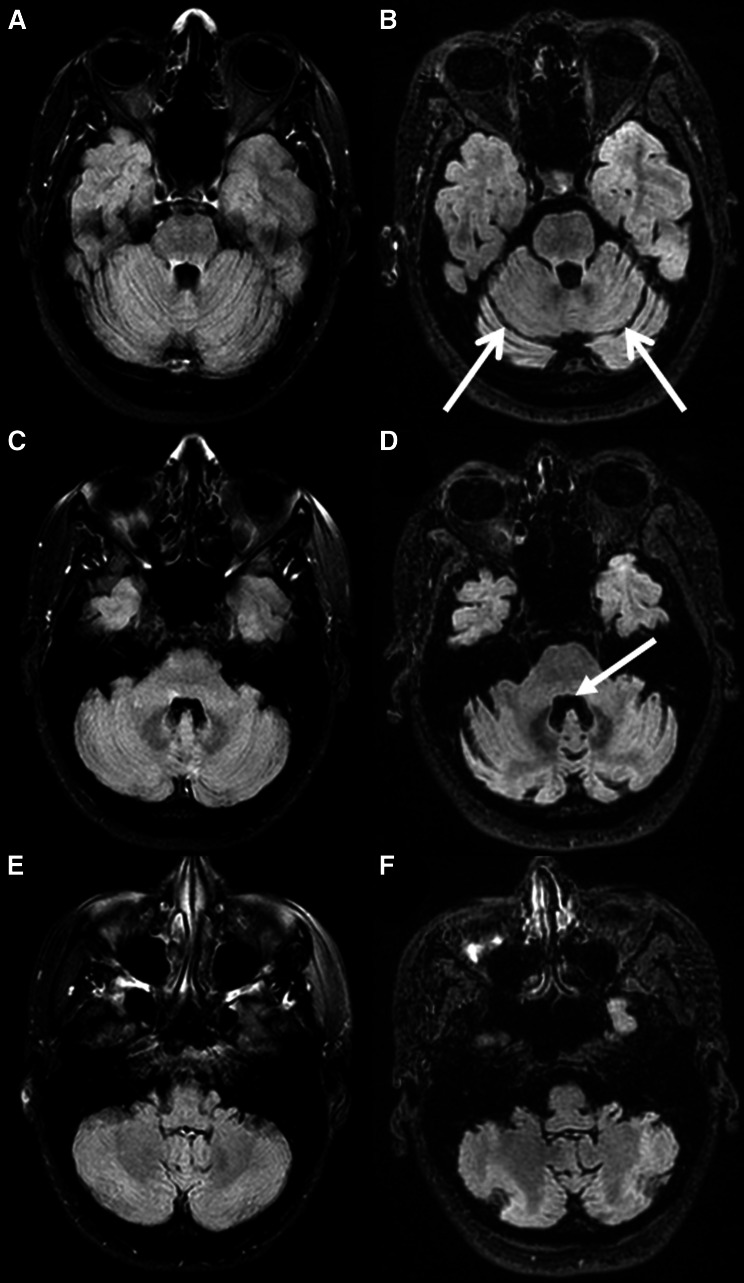
Axial fluid attenuated inversion recovery (FLAIR) images of patient 1 showing no cerebellar atrophy at the time of PML diagnosis (grade 0, **a**–**c**) and grade 2 cerebellar atrophy 3 months later (**d**–**f**). Note the bilateral dilated sulci (*open arrowhead*) in image **d** and the loss of cerebellar volume with enlargement of the fourth ventricle (*closed arrowhead*) in image **e**

Three patients in the PML group developed cerebellar atrophy or showed progressive cerebellar atrophy following PML diagnosis. The first patient had no cerebellar atrophy (grade 0) at the time of the diagnosis of supratentorial PML. A grade 2 cerebellar atrophy was detected at 3 months after PML diagnosis, without the occurrence of new cerebellar T2/FLAIR hyperintense lesions (Fig. 1). At PML diagnosis the patient presented with reduced ability to use her left hand and an impaired tandem gait. Two to three weeks after PML diagnosis the patient developed ataxic dysarthria and dysphagia, suggestive of cerebellar pathology.

**Fig. 2:**
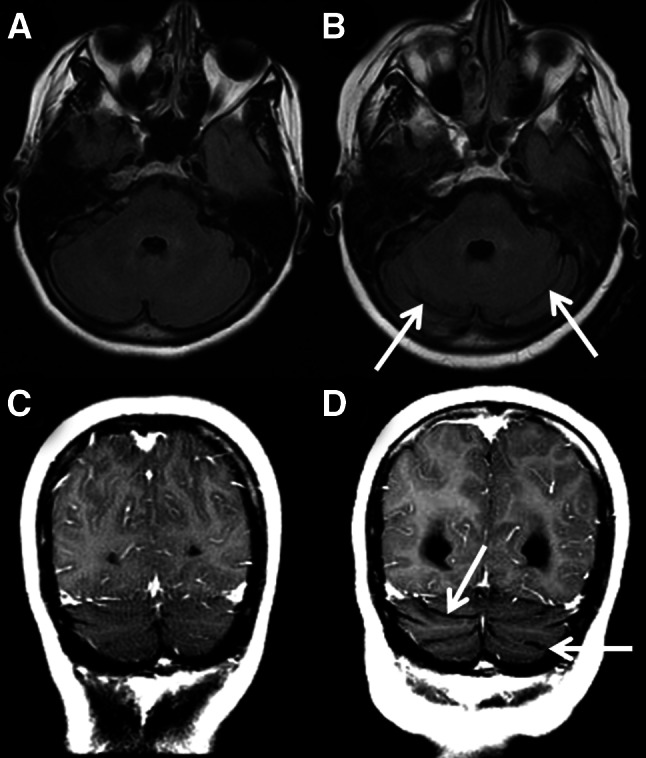
Axial fluid attenuated inversion recovery (FLAIR) images (**a**, **c**) and coronal T1 post-contrast images (**b**, **d**) of patient 2 showing no cerebellar atrophy at the time of PML diagnosis (grade 0, **a**, **b**) and grade 1 cerebellar atrophy 2.5 months later (**c**, **d**). Note the bilateral dilated sulci in image **c** and **d** (*open arrowheads*)

The second patient also had no cerebellar atrophy (grade 0) at the time of supratentorial PML diagnosis. However, grade 1 cerebellar atrophy was detected at 2.5 months after diagnosis, without new cerebellar T2/FLAIR hyperintense lesions (Fig. 2). Clinical information on the patient’s symptoms at the time of imaging could not be retrieved.

**Fig. 3:**
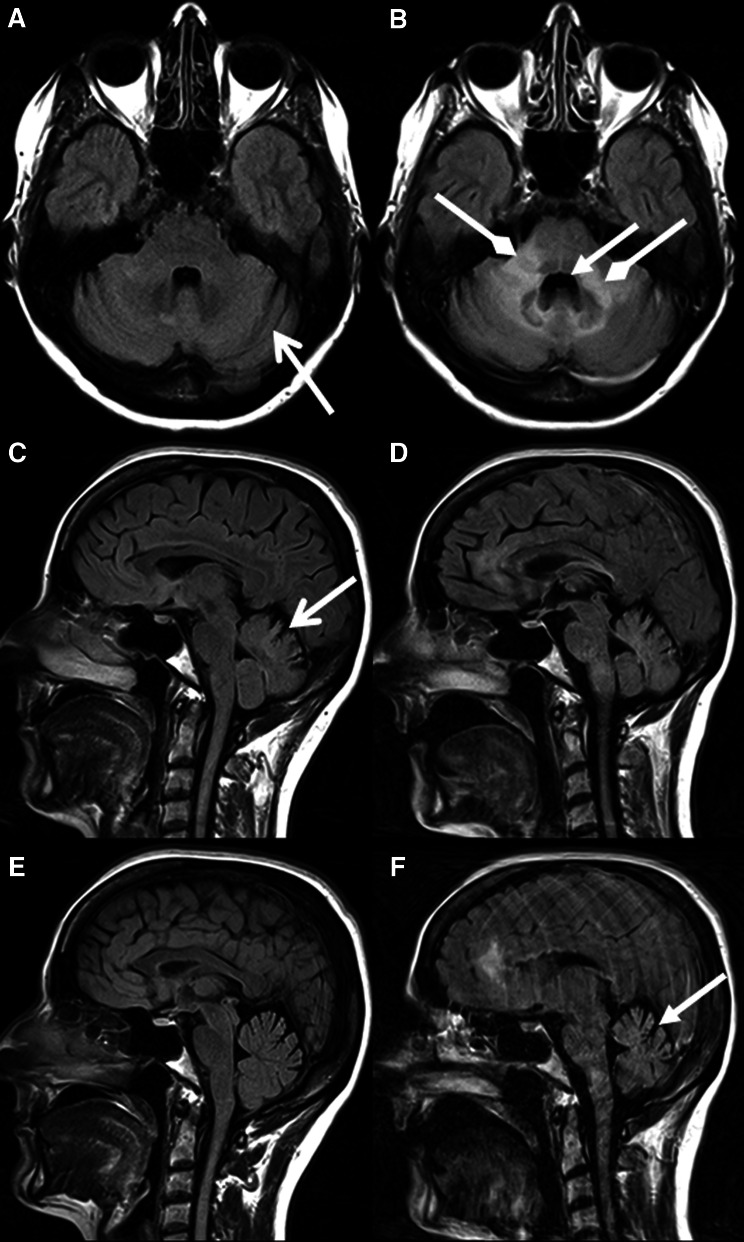
Axial fluid attenuated inversion recovery (FLAIR) images (**a**, **d**), and sagittal FLAIR images (**b**, **c**, **e**, **f**) of patient 3 showing grade 1 cerebellar atrophy at the time of PML diagnosis (**a**–**c**) and grade 2 cerebellar atrophy 3 months later (**d**–**f**). There were already bilateral dilated sulci at diagnosis (*open arrowhead*). However, there was clear loss of cerebellar volume 3 months later. Again, note the enlarged fourth ventricle (*closed arrowheads*). In addition, large hyperintense lesions in the cerebellar peduncles and brainstem developed (*diamond arrowheads*)

The third patient was diagnosed with a combination of supra- and infratentorial PML. Before and at the time of PML diagnosis a grade 1 cerebellar atrophy was present. Cerebellar atrophy progressed to grade 2, as there was clear loss of volume, at 3 months after diagnosis (Fig. 3). In addition, the bilateral cerebellar white matter lesions had increased in volume. At the time of PML diagnosis the patient presented with mild ataxia of her right arm and sub-acute slurred speech, suggestive of cerebellar pathology. Six weeks later the patient clinically deteriorated with severe ataxia, anarthria, visual loss and right-sided hemiparesis. These data are summarized in Table 3. In the other 8 patients with infratentorial PML lesions at the time of PML diagnosis and during follow-up, neither development nor progression of cerebellar atrophy was observed during follow-up. None of the patients in the control group showed any progression or development of cerebellar atrophy during the follow-up period.

**Table 3:** Degree of cerebellar atrophy and presence of infratentorial PML lesions in the three suspected GCN cases

	Atrophy grade before Dx	Atrophy grade at Dx	Atrophy grade after Dx	Months after Dx of scoring	“Infratentorial PML” lesions present?	Cerebellar symptoms
Patient 1	0	0	2	3 months	No	Yes
Patient 2	0	0	1	2.5 months	No	n/a
Patient 3	1	1	2	3 months	Yes	Yes

*PML* progressive multifocal leukoencephalopathy, *Dx* diagnosis of PML, *n/a* not available, four-grade cerebellar atrophy rating scale: grade 0: no atrophy; grade 1: dilated sulci; grade 2: loss of volume; grade 3: end-stage atrophy

## Discussion

Despite frequent MRI monitoring in natalizumab-associated PML patients, only two GCN cases associated with natalizumab treatment have been reported to date [12, 13]. As the imaging signs of GCN can be difficult to interpret, we hypothesized that this disease might be underdiagnosed in natalizumab-associated PML patients. In our series of 44 PML patients we indeed found three patients who developed cerebellar atrophy, or showed progression of already existing cerebellar atrophy, after PML diagnosis, suggesting concomitant presence of GCN.

In the first two patients cerebellar atrophy developed after a diagnosis of supratentorial PML, without the occurrence of new cerebellar white matter T2/FLAIR hyperintense lesions. Thus, this GCN imaging characteristic coincided with the manifestation of supratentorial PML, which in turn demonstrates active replication of JCV in the CNS of these two patients at that time. Therefore, we believe GCN is the most likely cause for cerebellar atrophy in these patients. The development of cerebellar symptoms in the first patient, weeks after PML diagnosis, further supports the suspicion of GCN in this patient. The third patient had pre-existing cerebellar atrophy which progressed after diagnosis of both supra- and infratentorial PML. One can debate whether the increase of cerebellar atrophy was the result of GCN or secondary to volume loss resulting from infratentorial PML manifestations. However, as none of the other eight patients with infratentorial PML developed cerebellar atrophy, we feel that this latter explanation is less likely, strongly suggesting GCN as the cause of the progression of the cerebellar atrophy. The cerebellar symptoms that this patient experienced could presumably be the result from both the new infratentorial PML lesions, as well as the rapidly progressive cerebellar atrophy.

MS pathology can also cause cerebellar atrophy, which explains the frequent cerebellar atrophy even before the occurrence of PML as seen in Table 2. However, the clear and rapid development or increase of cerebellar atrophy following PML diagnosis in our three GCN suspected patients, seems more than one would expect from the gradual neurodegeneration as seen in MS pathology. Indeed, none of the 25 patients in the control group showed any progression of cerebellar atrophy. The control group consisted of patients very similar to the PML group in terms of patient characteristics and was followed up for a period of about 1 year, which is much longer than the 2–3 months in which the suspected GCN patients showed progressive cerebellar atrophy.

The concomitant occurrence of GCN and PML has been reported previously in PML patients unrelated to natalizumab [10, 13, 14, 24]. Some of those patients had supratentorial PML and cerebellar atrophy, while others were diagnosed with infratentorial PML and showed progressive cerebellar atrophy. Approximately two-thirds of the reported GCN cases in the literature show additional white matter changes in the cerebellum [9].

It remains an interesting question whether GCN should be classified as a distinct disease entity or a variant of PML mainly affecting granule cells of the cerebellum. It has been shown that GCN is associated with certain mutations of the VP1 C terminus region of the JCV DNA. These JCV GCN mutants specifically infect cerebellar granule cell neurons and have been shown to have a disadvantage for growth in CNS white matter compared to wild-type JCV [25, 26]. In patients with both GCN and PML, coinfection of JCV with a GCN mutation and wild-type JCV has been shown [10]. Therefore, it is hypothesized that, at least in some cases with concomitant GCN and PML, wild-type JCV is responsible for the PML phenotype and JCV with VP1 mutations for the GCN phenotype [10].

Our study has several limitations. First, unfortunately there were no histopathological data available which could confirm our suspicion of GCN in the three suspected patients by showing infection of the granule cell neurons. Second, we were unable to recover CSF from the time of PML diagnosis of the three suspected GCN patients to identify possible JCV VP1 mutations. Finally, in most patients there was only one baseline scan present prior to PML diagnosis. This and the lack of JCV testing in CSF prior to the baseline scan precluded our ability to identify patients who had developed GCN prior to the baseline scan. Parameters related to image acquisition, in particular differences of pulse sequences (e.g., 2D versus 3D acquisition) and/or MR systems (e.g., 1.5 versus 3 T), might result in subtle differences in the visual appearance of cerebellar atrophy possibly leading to false-positive or false-negative results regarding the degree of cerebellar atrophy. We, therefore, confirmed any development or progression of cerebellar atrophy on subsequent scans obtained on MR systems and imaging protocols that were also used before the change in cerebellar atrophy.

With respect to natalizumab pharmacovigilance using MRI, the ability of MRI in prospectively identifying GCN in the absence of PML lesions remains questionable [18, 20], whether similar to the diagnostic dilemma in asymptomatic natalizumab-associated PML the detection of JCV DNA in the CSF can support the clinical and/or MRI diagnosis is unknown and needs to be investigated [27].

In conclusion, the identification of three probable GCN cases in natalizumab-treated MS patients with PML emphasizes the need for awareness of this combination or coincidence of different JCV-related diseases. Cerebellar symptoms in natalizumab-treated MS patients require early and frequent assessment for cerebellar atrophy, and if needed, JCV detection in CSF. In addition, assessment of (the progression of) cerebellar atrophy in the treatment of natalizumab-associated PML seems paramount.
